# Rapid Multiplex Antimicrobial Resistance Profiling and Bacterial Identification by LAP‐MALDI Mass Spectrometry Biotyping

**DOI:** 10.1002/advs.76927

**Published:** 2026-07-31

**Authors:** Lily R. Adair, Shabnam Iyer, Ian M. Jones, Rainer Cramer

**Affiliations:** ^1^ Department of Chemistry University of Reading Reading Berkshire UK; ^2^ Royal Berkshire NHS Foundation Trust Reading Berkshire UK; ^3^ School of Biological Sciences University of Reading Reading Berkshire UK

**Keywords:** antimicrobial resistance, clinical microbiology, LAP‐MALDI mass spectrometry, MALDI biotyping, top‐down proteomics

## Abstract

Rapid and accurate characterization of antimicrobial resistance is essential for effective patient treatment and outcomes. Infection‐causing microorganisms often harbour multi‐drug resistance, requiring multiple tests for identification. Here, we present a multiplex functional assay using liquid atmospheric pressure (LAP) matrix‐assisted laser desorption/ionization (MALDI) as next‐generation MALDI biotyping technology, which can accurately determine antibiotic resistance/susceptibility within three hours from <5 µL of bacterial culture, employing a beta‐lactam antibiotic panel. Strains with common resistance genes, including OXA‐48, KPC‐3, and VIM‐1, as well as susceptible isolates, are easily and reliably classified. Concurrent with multi‐drug testing, the same bacterial sample provides species‐identifying lipid and protein profiles (up to 100% classification accuracy) and characterization through tandem mass spectrometry (MS/MS) protein sequencing, facilitated by LAP‐MALDI's ability to generate multiply charged protein ions. Detection of multiple antibiotics, their degradation products and lipids/proteins with high mass accuracy, along with the possibility of protein sequencing, offers new diagnostic possibilities for clinical microbiology and antimicrobial stewardship that are less probability‐based than conventional MALDI biotyping.

## Graphical abstract

Liquid atmospheric pressure matrix‐assisted laser desorption/ionization (LAP‐MALDI) mass spectrometry (MS) provides multiplexed biotyping of microbial cultures using a fast and simple workflow. This is demonstrated for clinically relevant bacteria, resulting in accurate antimicrobial resistance (AMR) detection, speciation by MS/MS sequencing and conventional MALDI biotyping at the strain level using both protein and lipid MS profiles.

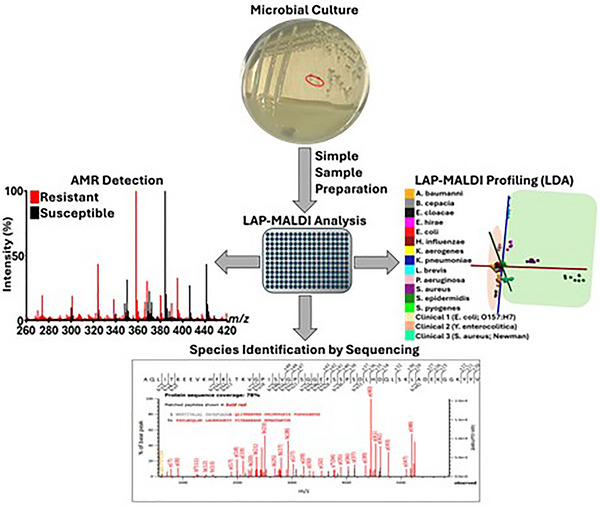

## 1 Introduction

Multidrug‐resistant (MDR) bacterial infections are a major global health threat, substantially contributing to morbidity and mortality worldwide with an estimated 4.71 million deaths associated with bacterial antimicrobial resistance (AMR) in 2021, of which 1.14 million were directly attributable to resistant infections [1]. The rapid and accurate identification of microbial pathogens is essential for guiding appropriate antimicrobial therapy, improving patient outcomes, mitigating the emergence of resistance, and reducing healthcare costs [2].

Bacterial infections are conventionally treated with targeted, pathogen‐specific antibiotics. However, their widespread misuse and overuse have driven the evolution of antibiotic‐resistant strains, rendering even last‐line treatments ineffective in severe cases [3]. Some of the most concerning MDR pathogens comprise the ESKAPE group — *Enterococcus faecium, Staphylococcus aureus, Klebsiella pneumoniae, Acinetobacter baumannii, Pseudomonas aeruginosa*, and *Enterobacter* species [4]. These bacterial pathogens exhibit a remarkable capacity for developing AMR, particularly through the production of β‐lactamases, conferring resistance to β‐lactam drugs such as carbapenems, often regarded as last‐resort treatments for MDR infections [5, 6].

However, four ESKAPE pathogens frequently exhibit carbapenem resistance (*Klebsiella pneumoniae, Acinetobacter baumannii, Pseudomonas aeruginosa*, and *Enterobacter* species) and are designated as priority pathogens for urgent research and intervention by the WHO [7]. Escalating resistance to cephalosporins, another class of β‐lactams, has further driven clinical reliance on carbapenems, and thus the emergence of carbapenemases and the dissemination of MDR pathogens [8].

The economic and clinical burden of AMR is staggering, with hospital costs in Europe alone exceeding €1.6 billion annually [9]. Projections suggest that by 2050, AMR could surpass all other causes of mortality worldwide [1]. These trends underscore the urgent need for innovative strategies enabling early and precise pathogen identification and (antibiotic‐specific) AMR detection.

Current clinical methods for AMR detection rely primarily on culture‐based assays, which assess phenotypic resistance by evaluating bacterial growth in the presence of antibiotics. However, these methods are inherently slow, requiring up to 72 h from sample collection to resistance reporting [10]. This delay often results in the administration of inappropriate antimicrobial therapy, which, if too broad, increases the risk of resistance selection, or if too narrow, leads to ineffective treatment [11, 12].

Molecular diagnostic approaches, such as polymerase chain reaction (PCR), offer more rapid and specific resistance detection by identifying single resistance genes directly from patient specimens [13]. Despite their speed, these assays are costly and require prior knowledge of the genetic target, limiting their applicability to narrow‐spectrum detection of specific gene variants. Furthermore, they fail to detect resistance mechanisms that are not genetically encoded, such as the upregulation of efflux pumps, and their cost remains a barrier to widespread clinical implementation [14, 15, 16]. Therefore, functional assays that directly determine the efficacy of antibiotic treatment are highly desirable.

Matrix‐assisted laser desorption/ionization (MALDI) time‐of‐flight (TOF) mass spectrometry (MS) has become a cornerstone of clinical microbiology, enabling rapid microbial species identification through proteomic profiling. More recently, MALDI‐TOF MS has been adapted for antimicrobial susceptibility testing (AST), demonstrating the potential to detect resistance‐associated markers in various bacterial species [17]. However, the absence of a comprehensive reference database cataloguing the masses of resistance markers for all pathogen‐drug combinations has impeded its clinical translation.

Other MALDI‐TOF MS‐based assays detect β‐lactamase activity by measuring the mass shift of antibiotics following β‐lactam hydrolysis, though these methods remain limited to single benchmark antibiotics [18].

The integration of machine learning (ML) with MALDI‐TOF MS presents another promising avenue for resistance prediction, leveraging computational algorithms to detect subtle spectral variations associated with AMR phenotypes [19, 20, 21]. ML‐based approaches have shown potential in predicting resistance to multiple antibiotics simultaneously, addressing the growing challenge of multidrug resistance [22]. However, these require extensive, high‐quality benchmark datasets and remain limited in their ability to detect novel resistance mechanisms.

Liquid Atmospheric Pressure‐MALDI (LAP‐MALDI) MS has been developed and optimized over the past decade [23], demonstrating significant success in the identification of clinically relevant bacteria, as well as in veterinary diagnostics [24, 25, 26]. Both applications leverage species‐ and class‐specific lipid and protein profiles for identification and classification.

In this study, we extend LAP‐MALDI's capabilities of highly accurate bacterial identification to multiplexed AMR detection, both within a single, rapid functional assay. This approach extends conventional MALDI biotyping by being able to record both lipid and protein profiles in the same spectrum with the option for bacterial protein sequencing. Furthermore, highly accurate multiplexed AMR testing by recording ion signals from individual antibiotics and their hydrolysis products with high sensitivity and mass accuracy adds to the many modalities of this simple sample workflow (for experimental details see references [27, 28] and Supporting Information).

## 2 Results and Discussion

To encompass a diverse range of bacterial species, this study included both Gram‐positive and Gram‐negative bacteria, as well as multiple members from the same genus. All thirteen NCTC species analyzed are clinically relevant and have the potential to exhibit AMR. Five of these were Gram‐positive bacteria, and for two species (*K. pneumoniae* and *E. coli*), antibiotic‐susceptible and resistant strains were analyzed. Additionally, three clinical isolates of originally unknown identity were also included in the study for double‐blinded validation and demonstrating the workflow's applicability to isolates beyond those from collections.

**FIGURE 1:**
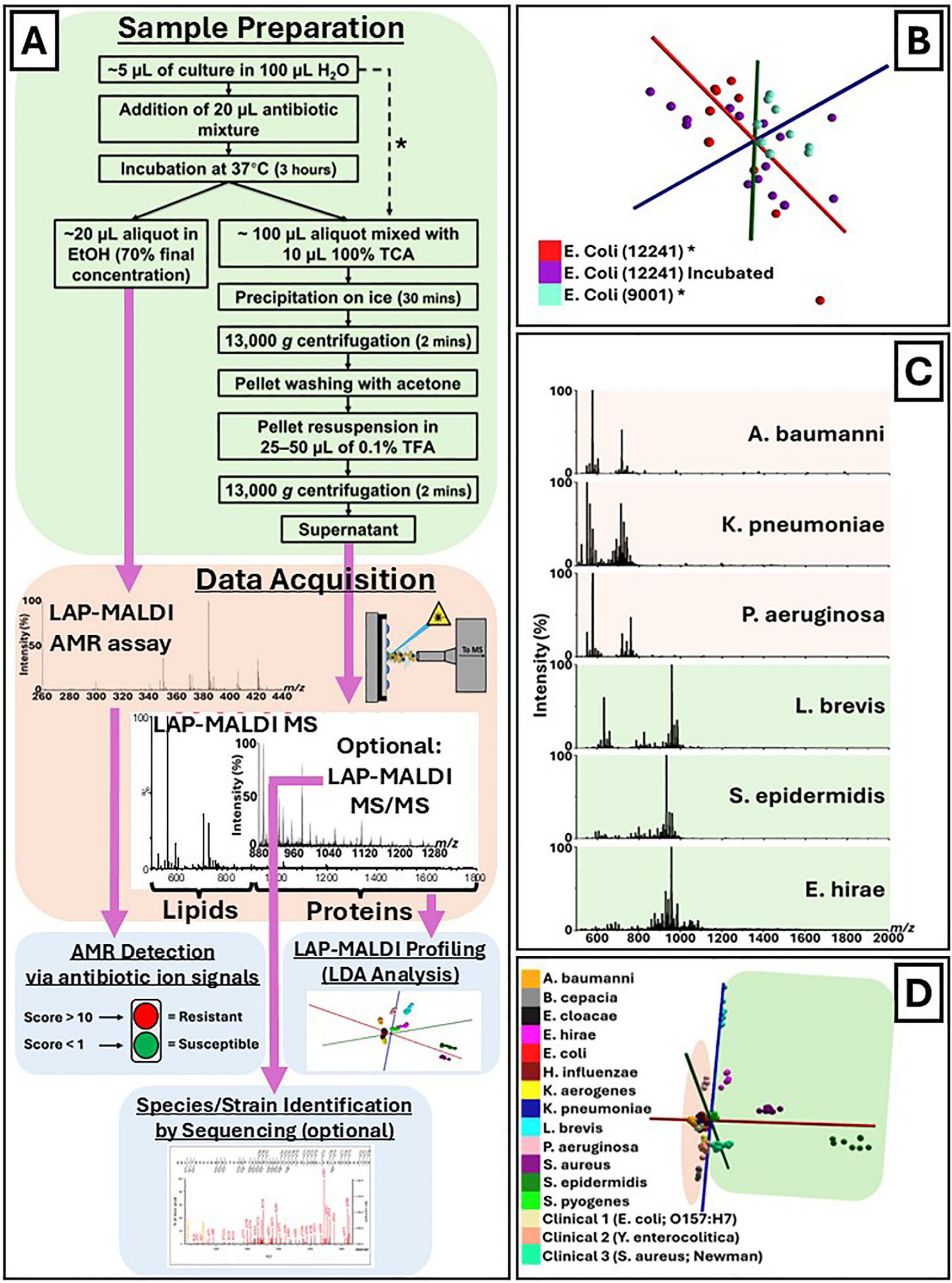
LAP‐MALDI MS Bacterial Culture Analysis. A, Overview of the workflow from bacterial culture and sample preparation to LAP‐MALDI MS data acquisition and analysis (* denotes the path for all susceptible strains, which followed the workflow without antibiotic incubation). B, PCA clustering of the LAP‐MALDI MS profiles (*m/z* range of 500–2000) of the two non‐resistant *E. coli* strains, of which one (NCTC 12241) was analyzed twice following two different workflow paths: with and without incubation (see the two alternative workflow paths for LAP‐MALDI MS profiling in panel A). C, Representative LAP‐MALDI MS spectra of lipid extracts from six bacterial species, including three Gram‐negative (salmon) and three Gram‐positive (green) species. D, Linear discriminant analysis (LDA) clustering of bacterial samples based on combined lipid and protein profiles (*m/z* range of 500–2000), from both treatments with and without antibiotics incubation. Gram‐negative species cluster in the salmon‐shaded space while Gram‐positive species cluster in the green‐shaded space.

A simple TCA extraction protocol was employed (see Experimental Details in Supporting Information and Figure 1A), enabling lipid/protein profiling within 40 min, from bacterial harvest to LAP‐MALDI MS data analysis. TCA precipitation was bactericidal, as shown by lack of culture following treatment, effectively removing operator risk for all subsequent steps.

In this study, all susceptible NCTC strains followed the dotted workflow line in Figure 1A (no incubation with antibiotics). All resistant strains as well as the two susceptible strains NCTC 9633 (*K. pneumoniae*) and NCTC 12241 (*E. coli*) were incubated with antibiotics. Thus, two data sets of LAP‐MALDI MS lipid/protein profiles were obtained for these two strains, which allowed an evaluation of MS profile changes induced by the addition of a 3‐h antibiotic incubation. The three clinical isolate strains (*E. coli* O157:H7 with Shiga toxin genes removed, *Y. enterocolitica*, and *S. aureus* (Newman)) were also incubated with antibiotics. Principal Component Analysis (PCA) of the LAP‐MALDI MS profile data for the susceptible *E. coli* strains (Figure 1B), of which NCTC 12241 was prepared with and without the 3‐h antibiotic incubation, and thus analyzed twice, showed no clear separation dependent on the strain or type of sample preparation. All replicates from both sample preparations were then combined and used for all speciation analyses. However, when the LAP‐MALDI MS profile data of the resistant *E. coli* strain was added to the above PCA analysis, separation was clear for the replicates between this strain and the susceptible strains (Figure S1).

### 2.1 LAP‐MALDI Biotyping Using Lipid and Proteoform Profiles

In general, LAP‐MALDI MS analysis generated unique lipid profiles in the *m/z* range of ∼500–1100 and protein profiles at *m/z* values above 1000. Clear visual distinctions between the MS profiles of Gram‐positive and Gram‐negative bacteria were obtained (Figure 1C).

The acquired MS ion signals were processed and binned (bin width of *m/z* 1), and the data was linearized based on PCA. Linear Discriminant Analysis (LDA) was then performed within the *m/z* range of 500–1100 and 500–2000, using the MS profile data of all 22 bacterial strains. Three‐dimensional LDA revealed distinct clustering of bacterial species and clear separation between Gram‐positive and Gram‐negative species for both *m/z* ranges (see Figure 1D for *m/z* 500–2000 and Figure S2 for *m/z* 500–1100). This correct separation included the three clinical isolates, of which two were Gram‐negative and one was Gram‐positive and whose identities were originally unknown.

For LDA using the *m/z* range of 500–2000, cross‐validation yielded a species classification accuracy of 98.9% (excluding outliers) using a 6‐standard deviation (SD) threshold. Including outliers, classification accuracy remained high at 96.0%. Limiting the analysis to the *m/z* range of 500–1100 and therefore focusing solely on the lipid profiles achieved a 100% classification accuracy when excluding outliers and a similar accuracy when including outliers (97.5%, with one fewer outlier).

### 2.2 LAP‐MALDI Biotyping/Speciation Using Top‐Down Proteoform Sequencing

**FIGURE 2:**
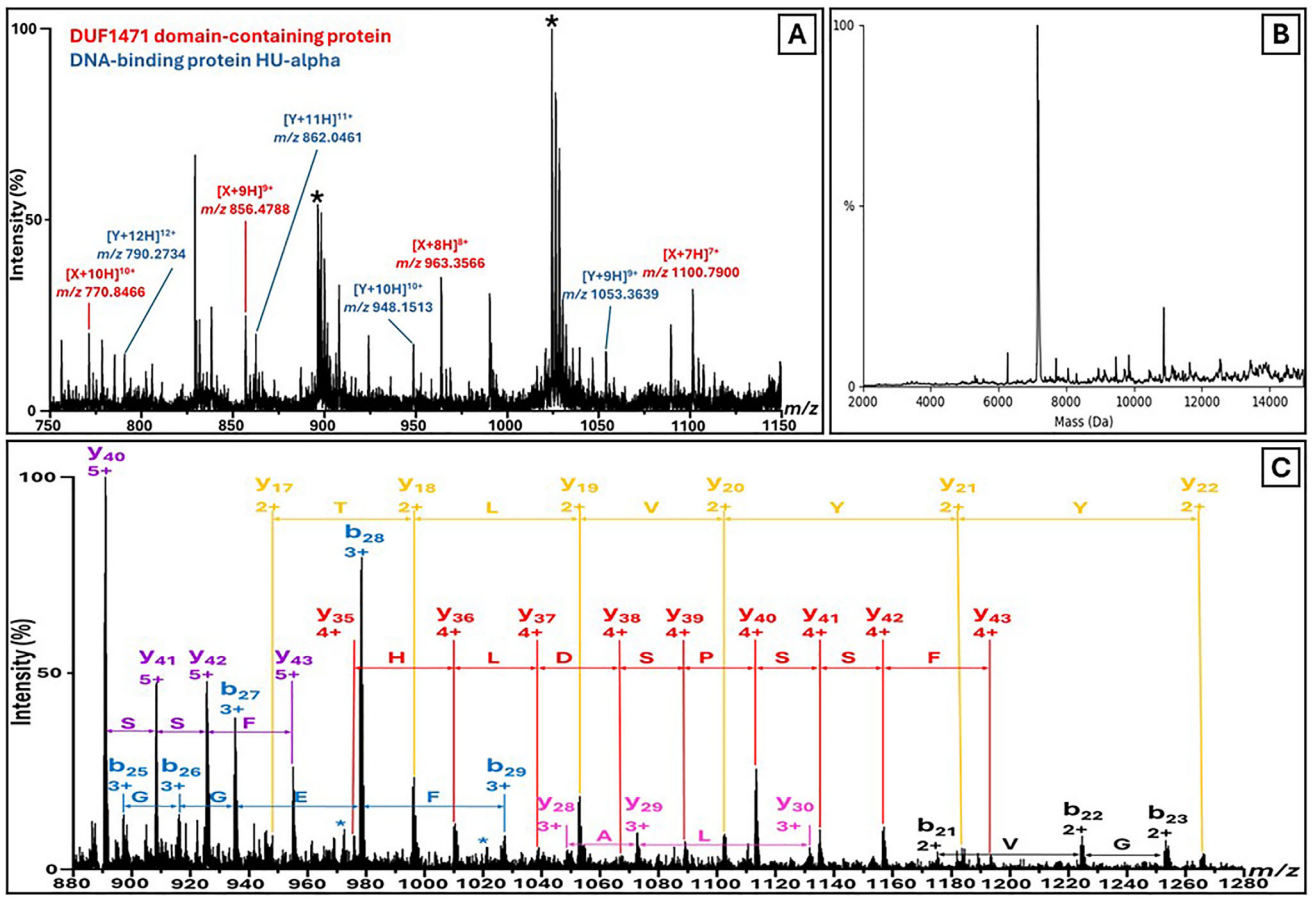
Bacterial Identification by LAP‐MALDI MS/MS Sequencing. A, LAP‐MALDI MS partial profile of *K. pneumoniae* (VIM‐1), detailing the proteome‐relevant *m/z* range. Peaks marked with * were previously identified by LAP‐MALDI MS/MS as *K. pneumoniae* proteoform of the major outer membrane lipoprotein Lpp. B, Deconvoluted spectrum of the MS profile partially shown in panel. C, LAP‐MALDI MS/MS spectrum of the proteoform with a mass of ∼7700 Da (refer to panels A/B), enabling species‐specific identification (DUF1471 domain‐containing protein).

LAP‐MALDI can further enhance confidence in species identification by utilizing LAP‐MALDI's ability to generate multiply charged ions, thus enabling its effective use for tandem mass spectrometry (MS/MS) analysis. Figure 2 displays a plethora of protein ion signals that were obtained by LAP‐MALDI MS and MS/MS analysis, using the example of *K. pneumoniae* (VIM‐1). MS/MS analysis identified a ∼9470‐Da proteoform of the DNA‐binding protein HU‐alpha (Figure S3), which is specific for the family Enterobacteriaceae, while MS/MS analysis of another proteoform with a mass of ∼7700 Da provided species‐specific identification (DUF1471 domain‐containing protein; Figure 2C and Figure S4).

### 2.3 Multiplexed Functional AMR Assay

LAP‐MALDI biotyping analysis was further extended by including individual antibiotic ion signals. For this analysis, the bacterial culture solution was incubated with an antibiotics mixture for three hours. Conversion to the hydrolyzed‐decarboxylated antibiotic product was observed as early as one hour (Figure S5), but three hours of incubation yielded the highest conversion.

Two antibiotic‐sensitive strains and five antibiotic‐resistant strains with resistance to some, if not all, β‐lactam antibiotics were analyzed for *E. coli* and *K. pneumoniae* as well as the three clinical isolates. β‐lactam antibiotics from five different classes (ampicillin, cefalexin, doripenem, imipenem and meropenem) were selected to create a clinically relevant antibiotic test mixture. Post incubation, a single‐step ethanol extraction was performed on a 20‐µL aliquot of the bacterial‐antibiotic suspension (whilst the remaining sample was subject to TCA precipitation), simultaneously inactivating the bacteria [29], an approach particularly useful for analysing highly pathogenic organisms. Direct TCA precipitation was unsuitable for resistance assessment due to acid‐induced antibiotic degradation (Figure S6), which could generate false‐positive resistance signals. Following ethanol inactivation, a simple centrifugation step collected insoluble material, and the supernatants were spotted 1:1 with the LAP‐MALDI matrix for MS analysis. Although the data was acquired manually, there is scope for automation and further reduction in analysis time [30, 31].

**FIGURE 3:**
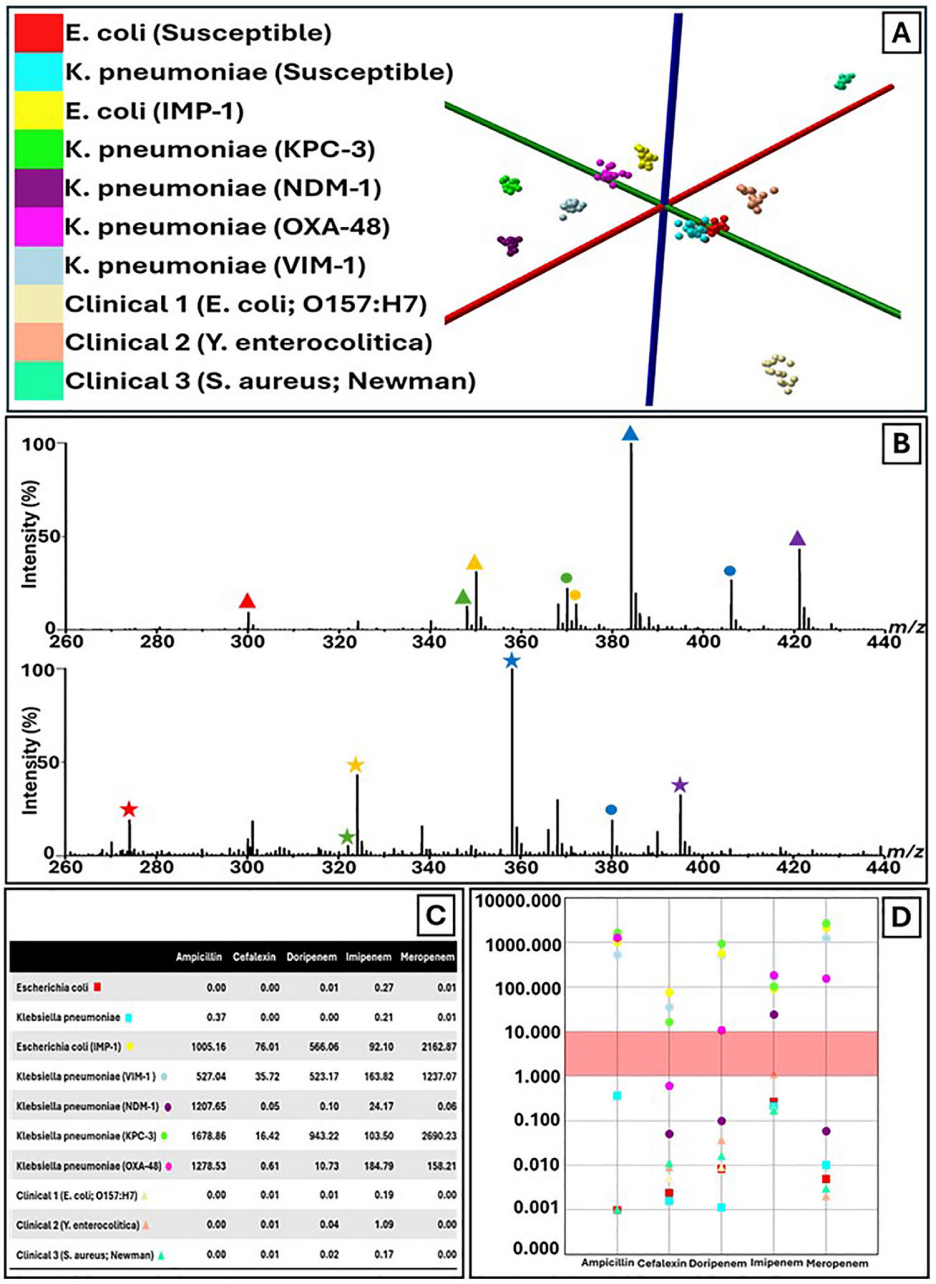
AMR Detection by LAP‐MALDI MS/MS Sequencing. A, LDA visualisation of lipid and protein profiles from each species used in the antibiotic assay, showing a clear separation between the five resistant and five susceptible strains. B, Zoomed‐in spectra of antibiotic profiles for susceptible *K. pneumoniae* (upper layer) versus *K. pneumoniae* VIM‐1 (lower layer), displaying intact antibiotics (▲) and their hydrolyzed decarboxylated products (★); additional sodiated antibiotic peaks are marked (⬤). C, Resistance scores calculated from a ratio of the intact antibiotic to hydrolyzed decarboxylated antibiotic. D, Resistance scores displayed on a logarithmic scale (strain coding as per panel C), highlighting a ‘dead‐zone’ where no resistance scores are observed, thus clearly distinguishing antibiotic susceptibility from resistance.

PCA/LDA analysis of the MS profiles (*m/z* 500–2000) from the samples incubated with antibiotics revealed clear discrimination between all *E. coli*, *K. pneumoniae* and the clinical strains, and separate clustering of the two major groups of antibiotic‐susceptible and resistant strains, respectively (Figure 3A).

LAP‐MALDI mass spectra of the ethanol‐inactivated aliquots revealed antibiotic‐related peaks in the *m/z* range of 260–440, with high mass accuracy and resolution (Figure 3B; Table S1). In antibiotic‐sensitive strains, the main ion signals corresponded to intact antibiotic species (ampicillin [M+H]^+^: *m/z* 350.12, cefalexin [M+H]^+^: *m/z* 348.10, doripenem [M+H]^+^: *m/z* 421.12, imipenem [M+H]^+^: *m/z* 300.10 and meropenem [M+H]^+^: *m/z* 384.16) (Figure 3B; upper spectrum), whereas for antibiotic‐resistant bacteria and in agreement with previous LAP‐MALDI studies [25], the hydrolyzed decarboxylated antibiotics were detected (ampicillin [M+H_2_O‐CO_2_‐H]^+^: *m/z* 324.14, cefalexin [M+H_2_O‐CO_2_‐H]^+^: *m/z* 322.12, doripenem [M+H_2_O‐CO_2_‐H]^+^: *m/z* 395.14, imipenem [M+H_2_O‐CO_2_‐H]^+^: *m/z* 274.13 and meropenem [M+H_2_O‐CO_2_‐H]^+^: *m/z* 358.18; see lower spectrum in Figure 3B). Besides accurate mass measurements (Table S1) and MS/MS analysis (e.g. Figure S7 for ampicillin), the assignments of these ion signals were also confirmed by analyzing penicillinase‐spiked susceptible strains incubated with these antibiotics, which provided the same antibiotic product ions as those obtained by incubation with the resistant strains (see Figure S8). The sodiated versions of the intact and hydrolyzed decarboxylated antibiotics can also be detected in certain instances (Figure 3B).

To assess the level of resistance exhibited by the bacteria, a resistance score was calculated based on the reciprocal ratio between the intact antibiotic and its hydrolyzed decarboxylated product ion signals (see Table S1 for *m/z* values). The score utilized the peak areas of the [M+H]^+^ ion signals as these were the most intense. All peaks were required to be within 25 ppm of the theoretical *m/z* value and to exhibit a signal‐to‐noise ratio (S/N) of >3. If there were no ion signals above the specified S/N within 25 ppm, a S/N value of 1.00 was assigned.

Calculated average resistance scores are in the range of 0–2690 (Figure 3C) with scores <1 indicating susceptibility as shown by the two susceptible strains with resistance scores between 0 and 0.37 for all antibiotics. Notably, for imipenem there was a single replicate for each susceptible strain where a S/N value of 1 was assigned due to insufficient ion signal of the intact imipenem. If these single replicates are omitted, the overall resistance scores are 0.08 for *E. coli* and 0.01 for *K. pneumoniae*.

Apart from strains NDM‐1 and OXA‐48, all resistant strains showed resistance scores of >16 for all antibiotics, i.e. approximately a 40‐fold difference to the susceptible strain scores. Importantly, the strains NDM‐1 and OXA‐48 both had a resistance score of <1 for cefalexin. For the NDM‐1 strain, the resistance scores for doripenem and meropenem were also well below 1, suggesting susceptibility to carbapenems.

For the clinical isolates, all isolates produced resistance scores that indicated susceptibility to all antibiotics. For Clinical Isolate 1, of which all samples were correctly classified as *E. coli* O157:H7 (Shiga toxin genes removed) in the MS profiling analysis, resistance to ampicillin has been previously reported in some cases, varying from below 10% to 100%, depending on the study. However, this phenotype was not observed in the present study. For Clinical Isolate 2, correctly classified as *Yersinia enterocolitica*, a resistance score of 1.09 was obtained for imipenem, placing it marginally within the proposed dead zone but displaying high susceptibility (low resistance scores) for all other antibiotics. Clinical Isolate 3 (*S. aureus*; Newman) showed similarly low resistance scores like Clinical Isolate 1, and therefore no resistance to any of the antibiotics tested.

In cases where the sodiated versions of the intact [M‐H+Na]^+^ and hydrolyzed decarboxylated [M+H_2_O‐CO_2_‐H+Na]^+^ antibiotics were present, they also followed the same trend.

### 2.4 Further Discussion

In this study, species classification was readily achieved through simple lipid profiling using LAP‐MALDI MS, with and without the additional use of proteoform profiling or tandem mass spectrometry (MS/MS), providing bacterial speciation with higher confidence.

In general, lipid MS profiles were consistently and reproducibly obtained as previously reported [32]. Lipids are key structural components of bacterial cell walls with clear differences between Gram‐negative and Gram‐positive organisms, consistent with the known structural divergence in their membrane lipid compositions. Gram‐negative bacteria possess an outer membrane rich in lipopolysaccharides (LPS) and phosphatidylethanolamines, contributing to a more diverse and abundant lipidome in the low *m/z* range. In contrast, Gram‐positive bacteria lack an outer membrane and instead feature a thick peptidoglycan layer embedded with lipoteichoic acids and primarily contain phosphatidylglycerol and cardiolipin as major lipid species [33]. These compositional differences result in characteristic LAP‐MALDI MS profiles that can reliably distinguish between bacterial species. Unlike conventional MALDI on axial TOF instruments, which is commonly used in clinical bacterial biotyping but suffers from high matrix ion interference and limited mass accuracy and resolution, LAP‐MALDI on Q‐TOF (or other orthogonal and high‐performance) instruments offers reduced interfering peaks and generally higher mass accuracy and resolution in the lower *m/z* range. This advantage enhances the detection of diagnostic lipid profiles and allows for the identification of individual lipids by accurate mass measurement and MS/MS analysis [25, 34].

For species classification by MS profiling, a multivariate model featuring 35 PCA dimensions and 11 LDA dimensions was applied. When comparing models using the full *m/z* 500–2000 range (lipid and protein profiles) versus *m/z* 500–1100 (lipid profiles only), all models provide between 98.5%–100% classification accuracy for all investigated SD (4‐9) when outliers are excluded. Outliers would be rerun or replaced with other replicates, and even when treating outliers as incorrect identifications, a classification accuracy of ∼95%–98% is obtained for both *m/z* ranges and all SD, apart from 4 SD when the accuracy dropped to around 90%. This overall stability reflects both the robustness of the classification model and the well‐clustered nature of the data. In classification analyses, outlier thresholds based on SD are commonly used but must be carefully balanced to avoid bias. Here, even at different thresholds, the model's performance remained largely unaffected, highlighting the reliability of LAP‐MALDI‐derived spectral features for bacterial classification.

Conventional MALDI‐TOF MS biotyping, as currently deployed in clinical laboratories, is well known for genus/species‐level identification but falls short in identifying diagnostic peptides or proteins due to the formation of predominately singly charged ions, leading in turn to poorer MS/MS performance. This limitation precludes direct proteoform identification and hinders structural elucidation of AMR‐associated biomarkers. In contrast, previous studies have already demonstrated that individual multiply charged proteinaceous ion species generated via LAP‐MALDI can be directly sequenced using top‐down proteomics approaches, further underscoring the technique's value in microbial biotyping [26, 35, 36]. LAP‐MALDI's ability to generate ‘ESI‐like’ multiply charged protein ions enables the employment of MS/MS proteoform sequencing, using high‐performance MS/MS instruments such as hybrid Orbitrap and Q‐TOF mass spectrometers.

To demonstrate this, selected multiply charged ions were subjected to collision‐induced dissociation (CID) MS/MS using the same liquid MALDI sample from which the MS profile was acquired. Species identification by top‐down CID MS/MS proteoform sequencing directly from LAP‐MALDI MS profile peaks was shown for *K. pneumoniae* (VIM‐1). While the MS/MS identification of the ∼9470‐Da proteoform was only specific for the Enterobacteriaceae family, the ∼7700‐Da proteoform was species‐specific.

Importantly, a proteoform at ∼7700 Da was previously identified as one of the 30 most impactful features for the detection of ceftriaxone‐resistant *K. pneumoniae* by machine learning in a study using 300,000 mass spectra from conventional MALDI biotyping, but its sequence and protein identity could not be obtained [19].

Current clinical MALDI MS biotyping could be potentially surpassed by using LAP‐MALDI and its protein sequencing capabilities, being more reliable than MS profiling analysis using class prediction models by adding more specificity via direct proteoform sequencing and sequence‐based speciation. Top‐down sequencing and thus confident proteoform identification of LAP‐MALDI MS profile ion signals can directly link mass spectral features to biologically and potentially clinically relevant agonists. Such functionality provides a significant advantage over current MALDI workflows.

Last but not least and most importantly, the LAP‐MALDI MS and MS/MS biotyping workflow can also integrate a multiplexed functional assay for AMR detection. The harvested bacterial material is simply incubated with a range of antibiotics and incubated at 37°C for 3 h (or potentially less). For LAP‐MALDI biotyping, this additional incubation has virtually no effect as shown by the high species classification accuracy using both MS profile data sets obtained with and without antibiotics incubation. Importantly, by taking out a small aliquot (∼20 µL) of the incubated samples before they are prepared for LAP‐MALDI speciation analysis, bacterial processing of antibiotics can be easily detected in the low *m/z* range and used for detecting AMR. Using data from samples with substantially different antibiotics and from species that cluster closely, this present study underscores the robustness, flexibility and breadth of such an assay for both bacterial identification and multiplexed AMR detection.

An antibiotic‐specific resistance score was established by utilizing the reciprocal ratio of the protonated intact antibiotic and its protonated hydrolyzed decarboxylated product. The antibiotic‐susceptible strains tested in this study showed a ratio value of well below 1 for all antibiotics tested, indicating good ion signal intensities for the intact antibiotics but virtually undetectable antibiotic products, while the resistant strains IMP‐1, VIM‐1, and KPC‐3 have scores well above 10, indicating far higher product than intact antibiotic ion signals and therefore clear evidence of resistance in these strains. This substantial gap in the resistance score values can be used to identify antibiotic‐specific resistance with high confidence, providing a functional multiplexed assay that can directly and accurately guide the specific type of antibiotic treatment.

Interestingly, for the OXA‐48 and NDM‐1 strains, the resistance scores for cefalexin were below 1, thus indicating susceptibility to cefalexin. OXA‐48 is a class‐D lactamase gene resulting in strong resistance to penicillins, but weaker hydrolysis activity against cephalosporins and carbapenems [37, 38, 39], in agreement with the calculated resistance score of 1278.53 for the OXA‐48 strain against ampicillin and much lower resistance scores for carbapenems and a score of 0.6 for the cephalosporin cefalexin. Furthermore, the data for the NDM‐1 strain suggest susceptibility to cefalexin, doripenem and meropenem, with scores of 0.1 or lower. Typically, *K. pneumoniae* strains producing OXA‐48 and/or NDM‐1 carbapenemases are resistant to a broad spectrum of β‐lactam antibiotics, including carbapenems like doripenem and meropenem [40]. However, certain isolates have exhibited susceptibility to these antibiotics, which can be attributed to factors such as the level of carbapenemase expression, the presence of additional resistance mechanisms, or the permeability of the bacterial cell membrane [41, 42, 43].

Considering the clear antibiotic‐specific separation between lactamase‐active and inactive strains, an adequately validated scoring system could be devised that rapidly provides antibiotic‐specific and clinically relevant information on the bacteria's AMR status, providing effective guidance on the use of antibiotics. As the data from this study show a clear ‘dead‐zone’ at the score range of 1–10 where no bacterium in this study produced a score (Figure 3C,D), a traffic‐light system can be envisaged with effective cut‐off threshold levels.

In this context, the many different analyses that can be accessed by LAP‐MALDI biotyping can further support AMR characterization as demonstrated by the detection and identification of the ∼7700‐Da isoform from the VIM‐1 strain. Since a proteoform was detected at this mass by conventional MALDI biotyping and previously assigned to the 30 most impactful features for the detection of ceftriaxone‐resistant *K. pneumoniae*, it could potentially serve as a proteomic marker for a cephalosporine‐resistant gene and therefore extend the application of LAP‐MALDI in AMR detection. As very little is currently known about this exact proteoform, additional work is needed to elucidate its wider role in AMR.

The low matrix cluster ion interference and high mass accuracy and resolution of LAP‐MALDI Q‐TOF MS are particularly advantageous in the low *m/z* range, where not only lipids but also smaller antibiotics and their degradation products are detected. Using an Orbitrap‐based LAP‐MALDI system [44] could further improve mass accuracy and resolution, particularly in the low *m/z* range. Reducing the time required for resistance profiling, potentially to as little as one hour, could further enhance clinical utility and improve patient outcomes.

## 3 Conclusion

Our study showcases LAP‐MALDI MS as a powerful tool for rapid and accurate bacterial identification and AMR characterization of clinically important and diverse bacterial strains, including antibiotic‐susceptible and resistant strains. As demonstrated with the employed set of isolates, the workflow is applicable to isolates from collections as well as those that are clinically obtained. The developed assay provides high mass accuracy and resolution, classifying bacteria with 100% accuracy and enabling multiplexed AMR analysis of antibiotic mixtures. Moreover, biotyping by MS profile analysis can be substituted (or supplemented) by proteoform sequencing, exploiting LAP‐MALDI's ability to generate multiply charged protein ions, and, therefore high‐quality MS/MS spectra on high‐performing hybrid mass analyzers. The integration of lipid/proteoform profiling, proteoform sequencing, and antibiotic profiling, all by LAP‐MALDI and within a single assay represents a major advancement for clinical microbiology. The ability to detect AMR within a short incubation period offers significant clinical advantages, potentially improving patient outcomes by expediting treatment decisions. This technology promises personalized precision medicine by optimizing antibiotic treatment as a fundamental pillar of effective antibiotic stewardship. By reducing the time required to obtain crucial, patient‐specific AMR information, LAP‐MALDI MS has great potential to ultimately improve patient outcomes, reduce unnecessary (broad‐spectrum) antibiotic use, and consequently, mitigate the development of AMR.

## Author Contributions

**Lily R. Adair**: data curation, investigation, methodology, writing – original draft, writing – review and editing, formal analysis. **Shabnam Iyer**: Writing – review and editing, validation, methodology, investigation. **Ian M. Jones**: writing – review and editing, validation, supervision, resources, methodology, investigation. **Rainer Cramer**: conceptualization, methodology, data curation, supervision, resources, formal analysis, validation, funding acquisition, writing – original draft, writing – review and editing, investigation, project administration.

## Conflicts of Interest

The authors declare no conflicts of interest.

## Supporting information




**Supporting File**: advs76927‐sup‐0001‐SuppMat.pdf.

## Data Availability

The data that support the findings of this study are openly available in the University of Reading Research Data Archive at https://doi.org/10.17864/1947.000468.
