# Supplementary material for: Rapid Multiplex Antimicrobial Resistance Profiling and Bacterial Identification by LAP‐MALDI Mass Spectrometry Biotyping
Source: Adv Sci (Weinh). 2026 Jul 31;13(60):e76927. doi: 10.1002/advs.76927 (PMC13427227; doi:10.1002/advs.76927)
Supplement: Supplementary file 1 [file ADVS-13-e76927-s001.pdf]

Supplementary Information

Rapid Multiplex Antimicrobial Resistance Profiling and Bacterial Identification by LAP-MALDI Mass Spectrometry Biotyping

Lily R. Adair[1], Shabnam Iyer[2], Ian M. Jones[3] and Rainer Cramer[1]*

[1]Department of Chemistry, University of Reading, Whiteknights, Reading, RG6 6DX, United Kingdom

[2]Royal Berkshire NHS Foundation Trust, London Road, Reading RG1 5AN, United Kingdom

[3]School of Biological Sciences, University of Reading, Whiteknights, Reading, RG6 6AJ, United Kingdom

*Address correspondence to:

Prof. Rainer Cramer, Department of Chemistry, University of Reading, Whiteknights, Reading RG6 6DX, UK.

Tel.: +44-118-378-4550; e-mail: r.k.cramer@reading.ac.uk

Table of Contents

Supplementary Experimental Details S3

Supplementary Figures S10

Supplementary Table S18

Supplementary Experimental Details

Reagents and Materials

First-generation NCTC (National Collection of Type Cultures) bacterial strains were purchased as freeze-dried discs from Pro-Lab Diagnostics (Wirral, UK). These included strains from *Lactobacillus brevis* (NCTC 13386), *Escherichia coli* (NCTC 12241and NCTC 9001), *Klebsiella pneumoniae* (NCTC 9633), *Pseudomonas aeruginosa* (NCTC 12903), *Staphylococcus aureus* (NCTC 6571), *Klebsiella aerogenes* (NCTC 9528), *Streptococcus pyogenes* (NCTC 12696), *Enterococcus hirae* (NCTC 13383), *Staphylococcus epidermidis* (NCTC 13360), *Burkholderia cepacian* (NCTC 10661), *Enterobacter cloacae* (NCTC 13380), Acinetobacter baumannii (NCTC 12156) and *Haemophilus influenzae* (NCTC 12975). Strains possessing antibiotic resistance and their respective resistance genes included *E. coli* (NCTC 13476, IMP-1) and four *K. pneumoniae* strains, *i.e.* NCTC 13440 (VIM-1), NCTC 13443 (NDM-1), NCTC 13438 (KPC-3) and NCTC 13442 (OXA-48).

The three clinical or near-clinical bacterial species/strains that were used were *Escherichia coli* O157:H7, *Yersinia enterocolitica* (O9) and *Staphylococcus aureus* strain Newman. The employed *E. coli* O157:H7 strain is a member of the verotoxin-producing strains of *E. coli* (VTEC) and was modified solely to remove the *stx* loci but retained other virulence features such as the ability to adhere to cell membranes (PMID: 15322044). The *Y. enterocolitica* (O9) strain is a serotype O9 isolate taken as a typical member of a laboratory collection of clinical specimens (PMID: 30294593). The *S. aureus* strain Newman was originally isolated from a case of osteomyelitis (PMID: 22442307) and is widely used as a clinically relevant isolate (PMID: 40341159;

40491039). The identities of these three strains were initially unknown apart from the fact that these were different to the NCTC strains used in this study.

α-Cyano-4-hydroxycinnamic acid (CHCA) was purchased from Bruker Daltonics (Coventry, UK). HPLC-grade water, along with laboratory reagent-grade acetone and ethanol (≥99%) were obtained from Fisher Scientific (Loughborough, UK). LC-MS-grade water and acetonitrile (ACN) were purchased from Honeywell (Bracknell, UK). Dehydrated nutrient agar and Columbia blood agar base culture medium were sourced from Oxoid (Thermo Fisher, Basingstoke, UK) while MRS Agar was acquired from Darwin Biological (Shawbury, UK). Defibrinated horse blood was supplied by EO Labs (Reading, UK). Ethylene glycol, trichloroacetic acid (TCA), ampicillin, cefalexin and penicillinase (from *Bacillus cereus*) were purchased from Sigma Aldrich (Gillingham, UK). Meropenem (trihydrate) and doripenem (monohydrate) were supplied by MedChemExpress through Fisher Scientific (Loughborough, UK). Imipenem (monohydrated) was obtained from Toronto Research Chemicals (Toronto, Canada).

Sample Preparation

First-generation bacterial strains were revived from freeze-dried discs according to the supplier's instructions. The bacterial strains were stored in 70% glycerol at -80°C until needed. All culture media were prepared following the supplier's instructions, including autoclaving at 121 ˚C for 22 minutes to ensure sterility.

For growth on solid media, a loopful of the glycerol stock was streaked onto the appropriate agar and incubated under the required conditions, which were obtained by referencing the catalogue number of the NCTC.

After growth, approximately 5 µL of biological material was resuspended in 100 µL of LC-MS grade water. Samples that did not undergo antibiotic (Ab) incubation proceeded directly to protein precipitation. For the bacterial suspensions of the susceptible strains of *K. pneumoniae* (NCTC 9633) and *E. coli* (NCTC 1224) as well as the resistant strains of *E. coli* (NCTC 13476, IMP-1) and *K. pneumoniae* (NCTC 13440, VIM-1; NCTC 13443, NDM-1; NCTC 13438, KPC-3 and NCTC 13442, OXA-48) and the three clinical isolates, an aliquot of 20 µL of an aqueous Ab mixture containing ampicillin, cefalexin, doripenem, imipenem, and meropenem (each at 4 mg/mL) was added. The suspensions of the susceptible strains were then incubated at 37°C for 3 hours with and without the addition of penicillinase at a final concentration of 1 mg/mL as a control. Following incubation, 20 µL of each suspension was transferred to a new microcentrifuge tube, and 47 µL of ethanol was added to achieve a final concentration of 70% (v/v) before analysis.

For protein precipitation, 10 µL of 100% TCA was added to the remaining volume of each suspension (100 µL) and left to precipitate on ice for 30 minutes. The samples were centrifuged at 13,000 *g* for 2 minutes, and the resulting pellet was washed with 100 µL of acetone. The supernatant was discarded, and the pellet was resuspended in 25–50 µL of 0.1% TFA, depending on the pellet size, followed by centrifugation at 13,000 *g* for 2 minutes. The resulting supernatant was used for further analysis.

LAP-MALDI Sample Droplet Preparation

A CHCA-based liquid MALDI matrix was used for all analyses and was prepared by dissolving CHCA in an acetonitrile:water mixture (7:3, v/v) to a final concentration of 15 mg/mL. After thorough vortexing, ethylene glycol was added at 70% volume and

vortexed again to ensure homogeneity. Matrix solution (500 nL) was then spotted onto a stainless-steel MALDI sample plate and mixed on target with 500 nL of bacterial extract through aspirating/dispensing, forming a 1-μL liquid droplet for each MALDI sample.

Replicates

For samples that were not incubated with Ab, three biological replicates were each prepared on a different agar plate and day. All biological replicates underwent the same analyte extraction, with each extract analyzed in three LAP-MALDI sample droplet (technical) replicates, totalling nine samples per bacterium.

For samples subjected to antibiotic incubation, five biological replicates were each prepared on a different agar plate and day. All biological replicates underwent the same analyte extraction and were analyzed without technical replicates.

The penicillinase assay was performed with three biological replicates, each prepared on a different agar plate and day and analyzed by LAP-MALDI MS in three technical replicates.

LAP-MALDI MS and MS/MS

Data acquisition was performed using a Synapt G2-Si instrument (Waters, Wilmslow, UK) equipped with an in-house-built AP-MALDI source. A detailed description of the LAP-MALDI source can be found in a previous publication [43]. A 343-nm UV laser (FlareNX 343-0.2-2; Coherent, Santa Clara, USA) operated at a pulse repetition rate of 50 Hz was used, with its beam focused onto the centre of the MALDI sample droplet, delivering approximately 10 μJ per pulse (3 ns) for desorption. All data were acquired with 'Mobility TOF' activated, as well as in positive ion and sensitivity mode. It is worth

noting that ‘Mobility TOF’ mode was not required but was used to demonstrate its additional benefits. The source was operated at an extraction potential of approximately 3 kV, with an N_2 counter-gas flow of 210 L/h applied to the ion transfer tube. The instrument was manually calibrated over an *m/z* range of 50−2000 using Intellistart software (MassLynx 4.2; Waters) by analyzing a 0.5-µL aliquot of 500 ng/µL caesium iodide in isopropanol:water (1:1, v/v) mixed directly on the MALDI sample plate with a 0.5-µL aliquot of the liquid MALDI matrix but without the matrix chromophore CHCA.

For MS/MS experiments, the *m/z* value of the target precursor ion was selected, and the quadrupole isolation window was adjusted by setting the low-mass (LM) and high-mass (HM) resolution values around the selected *m/z* value for collision-induced dissociation (CID) MS/MS. The LM and HM resolution values of the quadrupole were set within the range of 4.5–4.8 and 15–18, respectively. The trap collision energy for CID ranged from 15–60 V, depending on the *m/z* value and charge state of the precursor ion.

For all data acquisitions, the scan time was set to one second per scan. Each MS data acquisition lasted either 30 seconds for antibiotic (Ab) profiles or 60 seconds for lipid/protein profiles. MS/MS data acquisitions ranged from 30 to 300 seconds, depending on the analyte type (antibiotic or protein) and ion signal intensity.

Linear Discriminant Analysis (LDA)

Statistical analysis was conducted using the AMX Abstract Model Builder [Beta] version 1.0.2259.0 (Waters) to analyse combined lipid and antibiotic-resistant profiles. Spectra from all scans were merged into a single representative spectrum per sample, resulting in a total of 276 spectra consisting of 9 spectra per species, except for *E. coli* (48

spectra: 18 susceptible without antibiotics, 15 susceptible with antibiotics, and 15 resistant with antibiotics), *K. pneumoniae* (84 spectra: 9 susceptible without antibiotics, 15 susceptible with antibiotics, and 60 resistant with antibiotics) and the three clinical isolates (15 each with antibiotics).

For species identification using the lipid/protein profiles, the software binned the data at 1-Da intervals across the *m/z* range of 500–2000. This was then followed by principal component analysis (PCA) for dimensionality reduction and linear discriminant analysis (LDA). The number of PCA and LDA dimensions used was 35 and 11, respectively. Pre-processing steps included background subtraction and normalisation. Cross-validation was carried out using the built-in '20% out' method. Outlier detection was based on thresholds ranging from 4 to 9 standard deviations. Identified outliers were flagged as samples requiring re-analysis in a clinical laboratory setting.

For antibiotic resistance classification, 30 PCA and 4 LDA dimensions were selected, using the same data binning and m/z range as for species identification.

Calculation of Resistance Scores

Ion signal-to-noise ratio (S/N) values were determined using Mascot Distiller (Version 2.8.5.1, 64-bit; Matrix Science, London, England) from automated peak picking. For 'MS Peak Picking', a correlation threshold (Rho) of 0.7 and a minimum S/N of 3.1 were applied. Baseline correction was performed using the isotope distribution fit method, with a maximum of 500 peak iterations per scan. Peak width parameters were set to a minimum peak width of 0.005 Da, expected peak width of 0.05 Da, and maximum peak width of 0.5 Da. Spectra from all scans were combined into a single spectrum per sample.

To calculate the resistance score, the S/N value of the hydrolysed decarboxylated antibiotic ion was divided by the S/N value of the intact antibiotic ion. If either peak was not detected, due to an S/N below 3.1 or an *m/z* deviation greater than ±25 ppm from the theoretical mass, a default value of 1.00 was assigned. The final resistance score for each strain was calculated using the mean intensity values from five biological replicates.

Proteoform analysis using MS/MS data searching

Raw data files were analysed using Mascot Distiller (Version 2.8.5.1, 64-bit; Matrix Science, London, England) with automated peak picking. For ‘MS Peak Picking’, a correlation threshold (Rho) of 0.7 and a minimum S/N of 3.1 were applied. Baseline correction was performed using the isotope distribution fit method, allowing a maximum of 500 peak iterations per scan. Peak width parameters were set to a minimum of 0.005 Da, an expected width of 0.05 Da, and a maximum of 0.5 Da. The exported peak list consisted of the monoisotopic masses of singly charged fragment ion equivalents.

MS/MS data were processed as described previously [44]. In brief, fragment ion signals from each MS/MS acquisition, originating from the same charge state distribution, were grouped within an *m/z* tolerance of ±0.1. Peaks were filtered using a minimum intensity threshold of 10 and required to appear in the MS/MS spectra of at least two different charge states. The resulting peak list was searched using Mascot’s MS/MS Ions Search (version 3.1; Matrix Science) against both the Mascot-provided contaminants database (20 Jan 2025; 247 sequences; 128,130 residues) and the trEMBL database (22 May 2024; 248,234,451 sequences; 87,367,689,973 residues), restricted to the taxonomy

'Bacteria'. Search parameters were defined as follows: mass values set to 'Monoisotopic', precursor ion tolerance of 20 ppm, and fragment ion tolerance of 0.2 Da. The instrument type selected was 'MALDI-QIT-TOF', and 'Oxidation (M)' was specified as a variable modification. For the initial searches, 'None' was selected for the enzyme parameter. If no significant matches were found (likely due to the large search space) the enzyme parameter was changed to 'NoCleave'.

Supplementary Figures

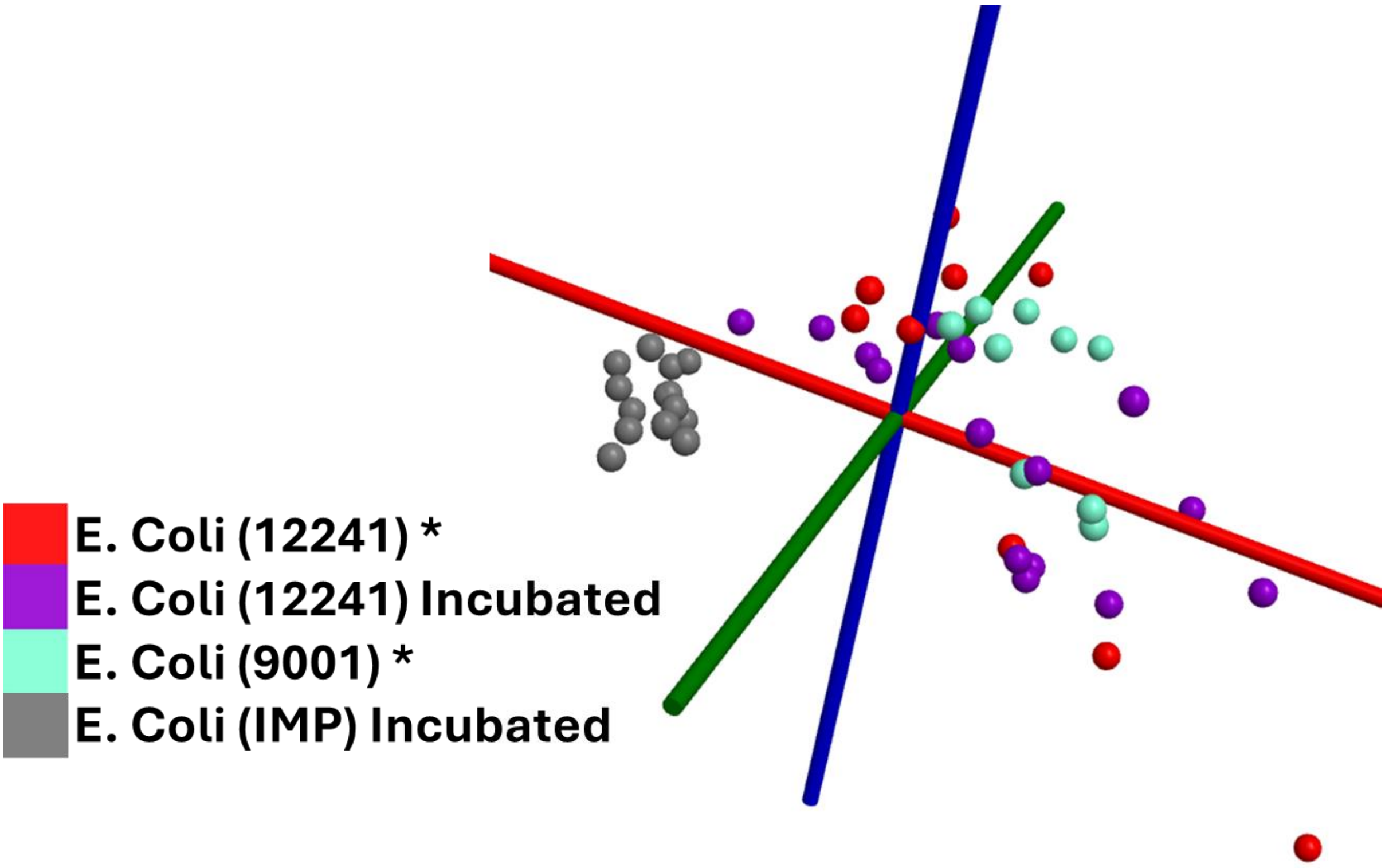

Figure S1 – PCA of the LAP-MALDI MS profiles (*m/z* range of 50-2000) showing no specific clusters for the non-resistant *E. coli* strains but separation of the resistant *E. coli* strain cluster from the non-resistant *E. coli* strains, of which one (NCTC 12241) was analyzed twice following two different workflow paths: with and without incubation (for details see Figure 1A and text).

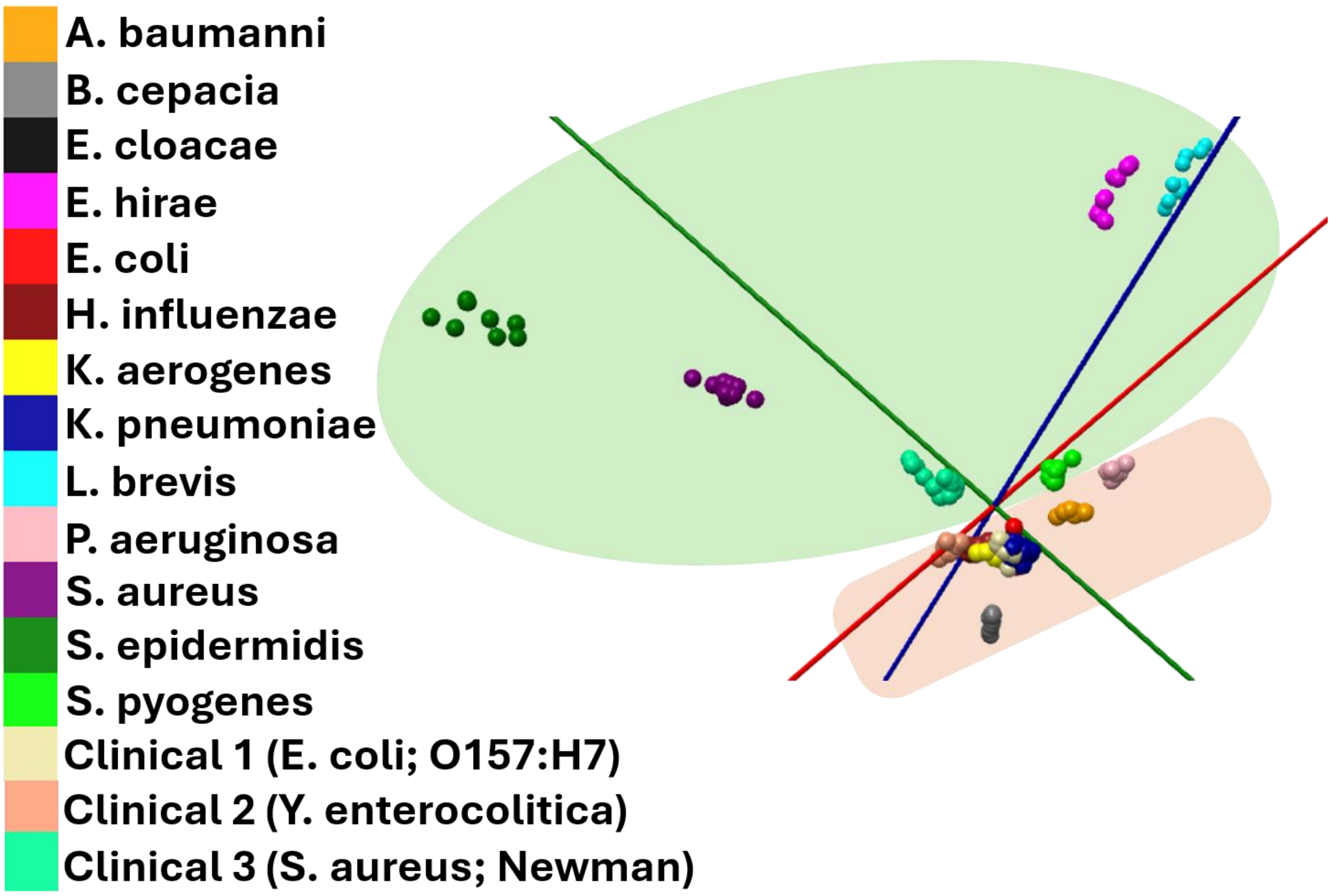

Figure S2 – PCA/LDA based on lipid profiles (*m/z* 500-1100) alone, including untreated and antibiotic-treated/incubated NCTC bacterial samples and antibiotic-treated clinical samples as well as all outliers. Gram-negative species cluster in the salmon-shaded space while Gram-positive species cluster in the green-shaded space.

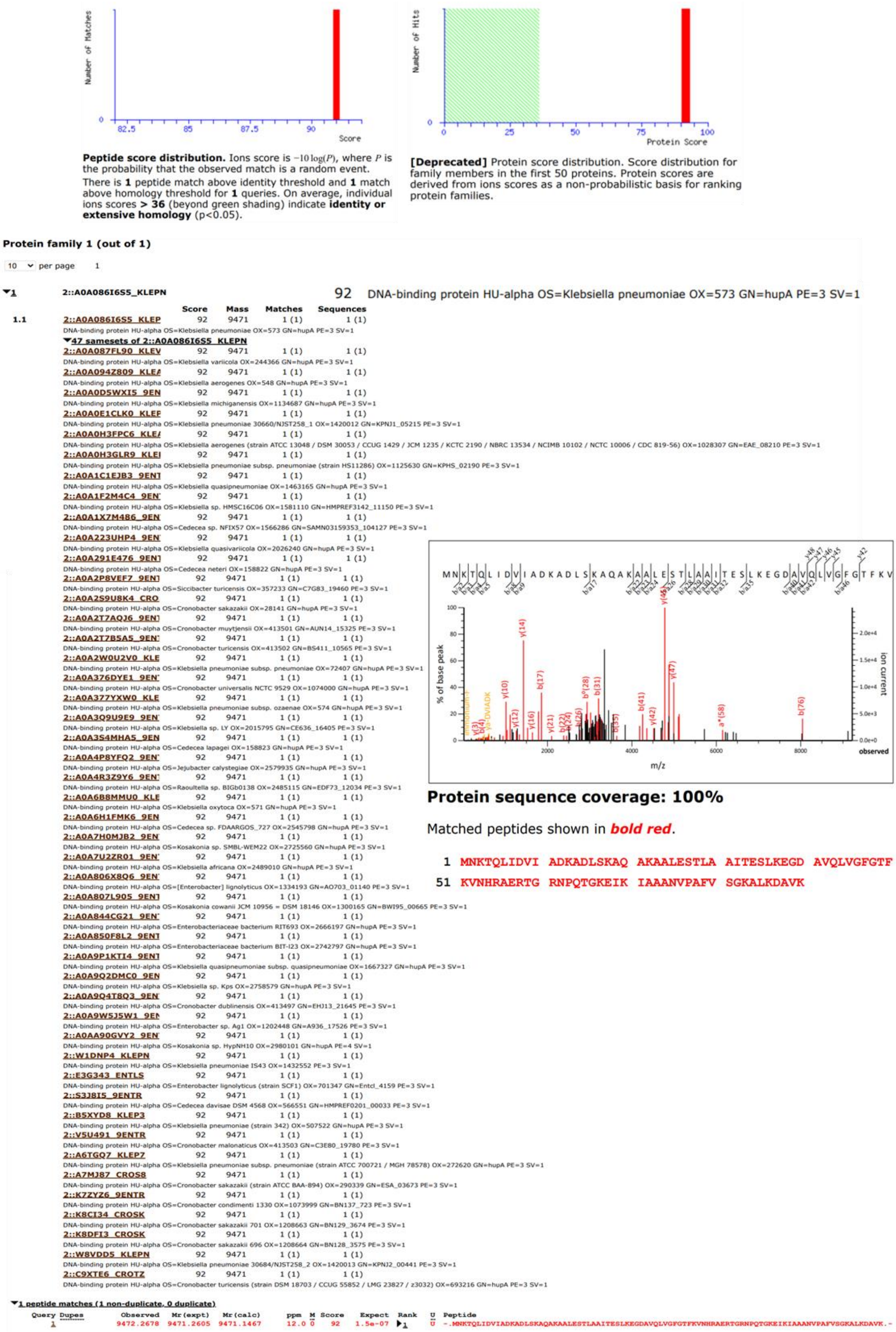

Figure S3 – Mascot search results identifying the proteoform with a mass of ~9,470 Da as DNA-binding protein HU-alpha.

▼Score distribution

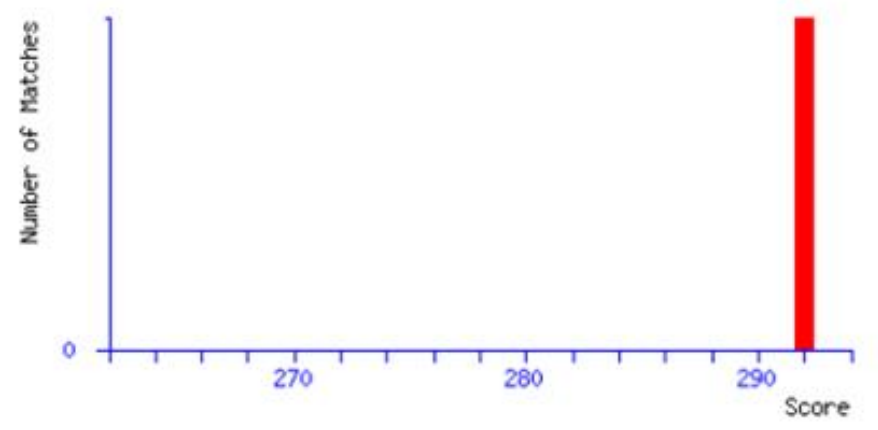

Peptide score distribution. Ions score is $-10\log(P)$, where P is the probability that the observed match is a random event.

There is **1** peptide match above identity threshold and **1** match above homology threshold for **1** queries. On average, individual ions scores **> 49** (beyond yellow shading) indicate **significant homology**, while individual ions scores **> 86** (beyond green shading) indicate **identity or extensive homology** (p<0.05).

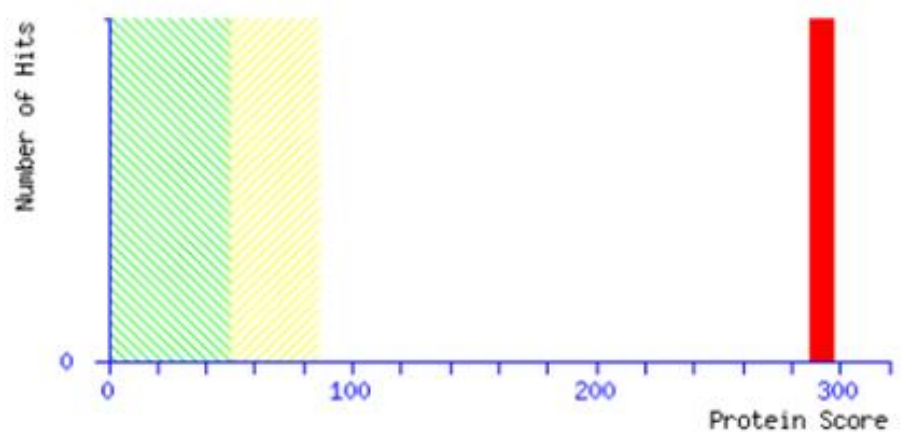

[Deprecated] Protein score distribution. Score distribution for family members in the first 50 proteins. Protein scores are derived from ions scores as a non-probabilistic basis for ranking protein families.

Protein family 1 (out of 1)

10 per page 1

▼1 **2::A0A087FU90_KLEVA** 292 DUF1471 domain-containing protein OS=Klebsiella variicola OX=244366 GN=AN2335V1_2139 PE=4 SV=1

		Score	Mass	Matches	Sequences
1.1	2::A0A087FU90_KLEV	292	9527	1 (1)	1 (1)
	DUF1471 domain-containing protein OS=Klebsiella variicola OX=244366 GN=AN2335V1_2139 PE=4 SV=1				
	**▼18 samesets of 2::A0A087FU90_KLEVA**				
	2::A0A0E1CHX1_KLEI	292	9527	1 (1)	1 (1)
	YdgH/BhsA/McbA-like domain-containing protein OS=Klebsiella pneumoniae 30660/NJST258_1 OX=1420012 GN=KPNJ1_04116 PE=4 SV=1				
	2::A0A0H3GT82_KLEI	292	9527	1 (1)	1 (1)
	YdgH/BhsA/McbA-like domain-containing protein OS=Klebsiella pneumoniae subsp. pneumoniae (strain HS11286) OX=1125630 GN=KPHS_13060 PE=4 SV=1				
	2::A0A1F2M9U2_9EN	292	9527	1 (1)	1 (1)
	YdgH/BhsA/McbA-like domain-containing protein OS=Klebsiella sp. HMSC16C06 OX=1581110 GN=HMPREF3142_06415 PE=4 SV=1				
	2::A0A1Y0Q3K0_KLEI	292	9527	1 (1)	1 (1)
	DUF1471 domain-containing protein OS=Klebsiella pneumoniae subsp. pneumoniae OX=72407 GN=E9161_10165 PE=4 SV=1				
	2::A0A2N4VZ74_9EN	292	9527	1 (1)	1 (1)
	DUF1471 domain-containing protein OS=Klebsiella quasipneumoniae OX=1463165 GN=C2767_20750 PE=4 SV=1				
	2::A0A377Z7M3_KLEI	292	9527	1 (1)	1 (1)
	Exported protein OS=Klebsiella pneumoniae subsp. ozaenae OX=574 GN=NCTC10313_01963 PE=4 SV=1				
	2::A0A486D4T2_KLEF	292	9555	1 (1)	1 (1)
	Exported protein OS=Klebsiella pneumoniae OX=573 GN=SAMEA4873652_01610 PE=4 SV=1				
	2::A0A486DJG0_KLEF	292	9587	1 (1)	1 (1)
	Exported protein OS=Klebsiella pneumoniae OX=573 GN=SAMEA4873656_01500 PE=4 SV=1				
	2::A0A5E5TMA4_9EN	292	9527	1 (1)	1 (1)
	Exported protein OS=Klebsiella quasivariicola OX=2026240 GN=SAMEA3538780_02069 PE=4 SV=1				
	2::A0A7S9HEW6_KLE	292	9771	1 (1)	1 (1)
	DUF1471 domain-containing protein OS=Klebsiella pneumoniae subsp. pneumoniae OX=72407 GN=IUJ34_06265 PE=4 SV=1				
	2::A0A7U3F2R1_9EN	292	9527	1 (1)	1 (1)
	YdgH/BhsA/McbA-like domain-containing protein OS=Klebsiella africana OX=2489010 GN=SB5857_01022 PE=4 SV=1				
	2::A0A7X1HWT5_KLE	292	9771	1 (1)	1 (1)
	DUF1471 domain-containing protein OS=Klebsiella pneumoniae OX=573 GN=H7U16_09620 PE=4 SV=1				
	2::A0A9P1P2K0_9EN	292	9527	1 (1)	1 (1)
	YdgH/BhsA/McbA-like domain-containing protein OS=Klebsiella quasipneumoniae subsp. quasipneumoniae OX=1667327 GN=KAM644c_36960 PE=4 SV=1				
	2::A0A9Q2HTM7_9EN	292	9527	1 (1)	1 (1)
	DUF1471 domain-containing protein OS=Klebsiella sp. Kps OX=2758579 GN=H8J56_24440 PE=4 SV=1				
	2::W1DFF6_KLEPN	292	9527	1 (1)	1 (1)
	Probable secreted protein OS=Klebsiella pneumoniae IS43 OX=1432552 PE=4 SV=1				
	2::B5Y0A7_KLEP3	292	9527	1 (1)	1 (1)
	YdgH/BhsA/McbA-like domain-containing protein OS=Klebsiella pneumoniae (strain 342) OX=507522 GN=KPK_4095 PE=4 SV=1				
	2::W8ULY4_KLEPN	292	9527	1 (1)	1 (1)
	YdgH/BhsA/McbA-like domain-containing protein OS=Klebsiella pneumoniae 30684/NJST258_2 OX=1420013 GN=KPNJ2_04139 PE=4 SV=1				
	2::W9BNK2_KLEPN	292	9527	1 (1)	1 (1)
	DUF1471 domain-containing protein OS=Klebsiella pneumoniae OX=573 GN=B5L96_00135 PE=4 SV=1				

▼1 peptide matches (1 non-duplicate, 0 duplicate)

Query	Dupes	Observed	Mr(expt)	Mr(calc)	ppm	M	Score	Expect	Rank	U	Peptide
1		7699.8528	7698.8455	7698.9638	-15.4	0	292	2.9e-26	▶1	U	A.AQLITKEEVKHFKLTKVGPISVGPSGGEFSSPSDLHDQLSKLADEKGGKYYVITAAREHGPNFEATAEVYK.-

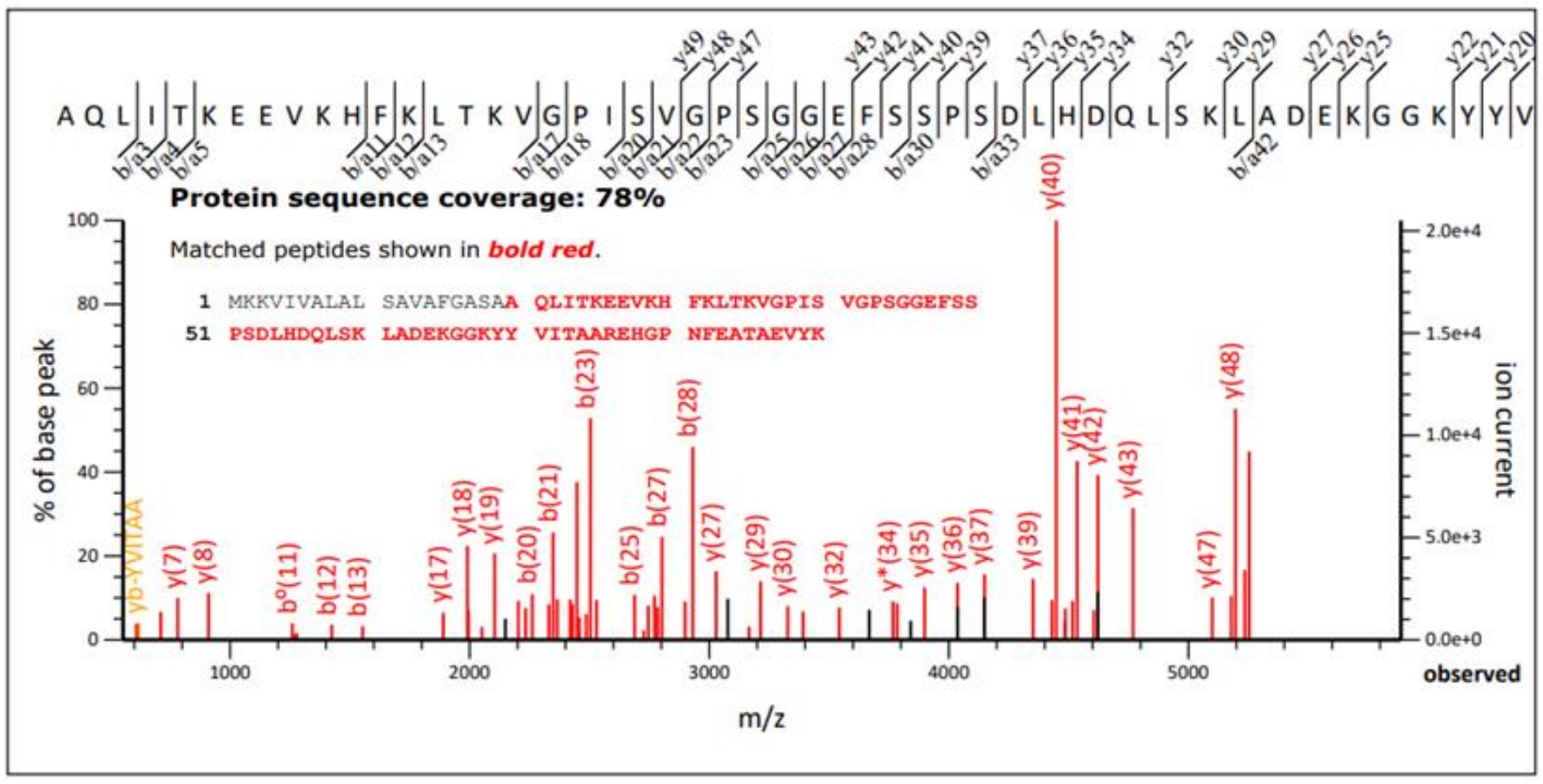

Figure S4 – Mascot search results identifying the proteoform with a mass of ~7,700 Da as a species-specific proteoform of the DUF1471 domain-containing protein.

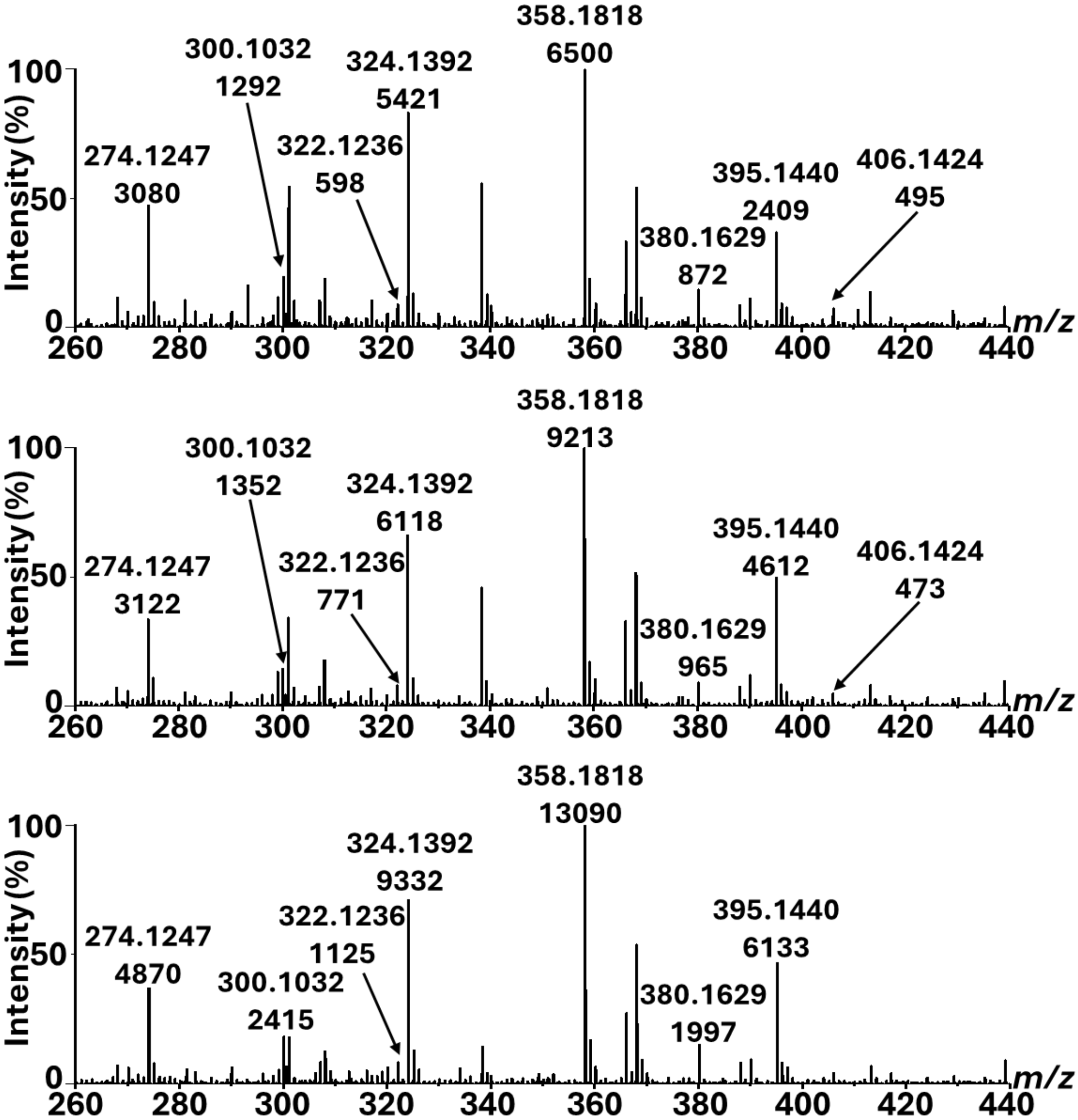

Figure S5 – MS spectra of *E. coli* (IMP-1) incubated with antibiotics: (top panel) incubation with antibiotics for one hour; (middle panel) incubation with antibiotics for two hours; (bottom panel) incubation with antibiotics for three hours.

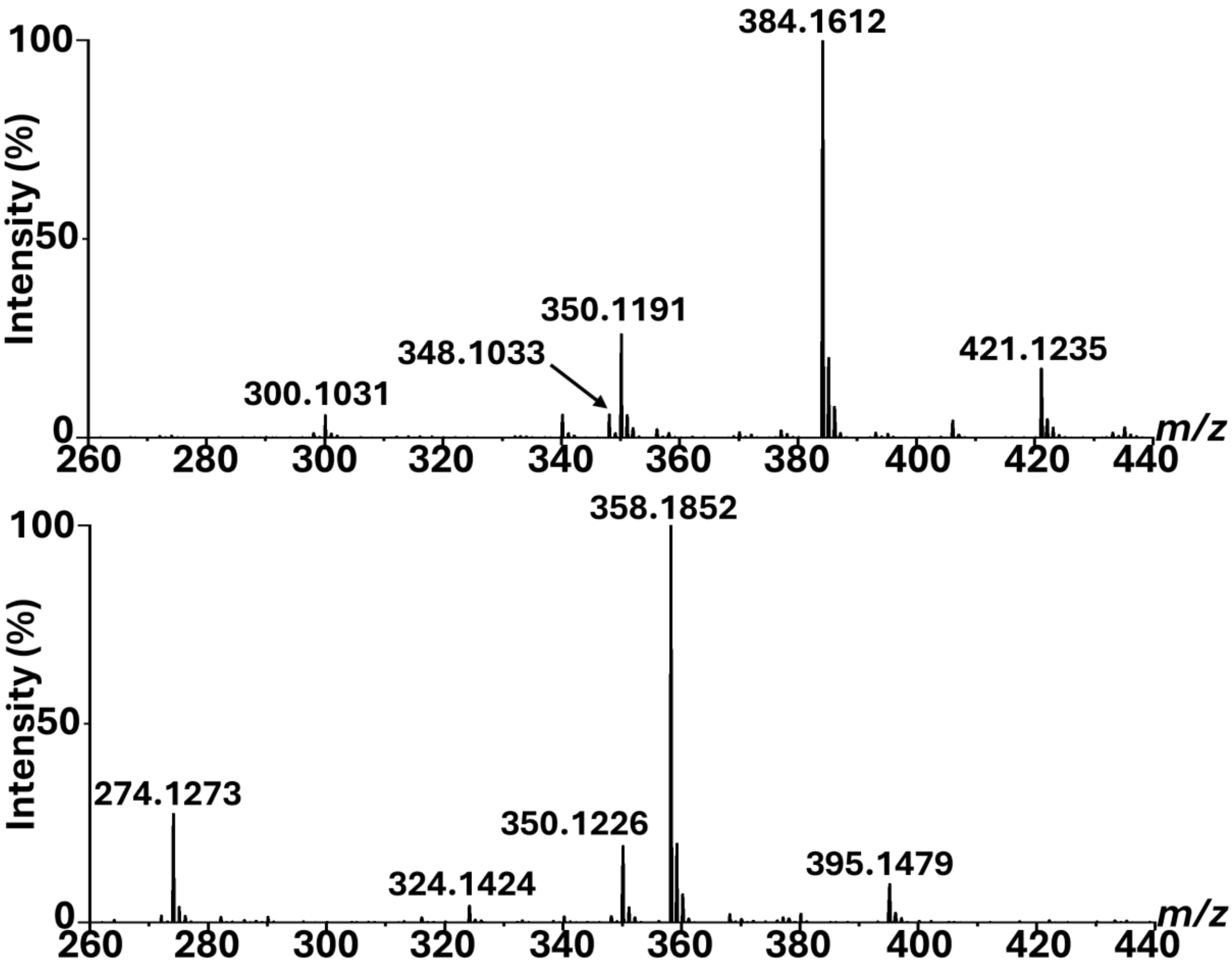

Figure S6 – MS spectra of the pure antibiotics mixture without bacteria incubated for three hours without TCA precipitation (top panel) and with TCA precipitation (bottom panel).

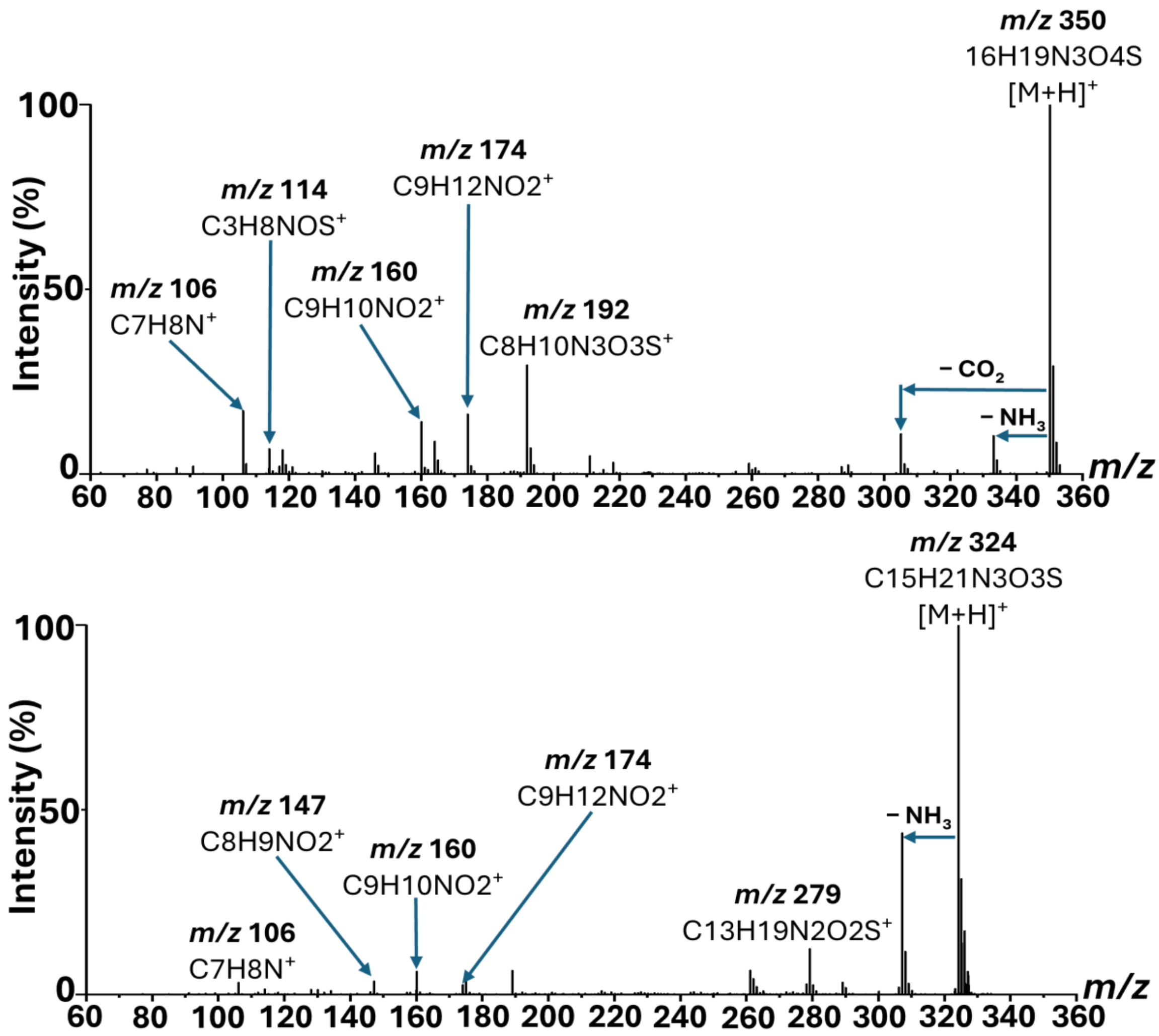

Figure S7 – LAP-MALDI MS/MS spectra of intact ampicillin (top panel) and hydrolyzed decarboxylated ampicillin (bottom panel).

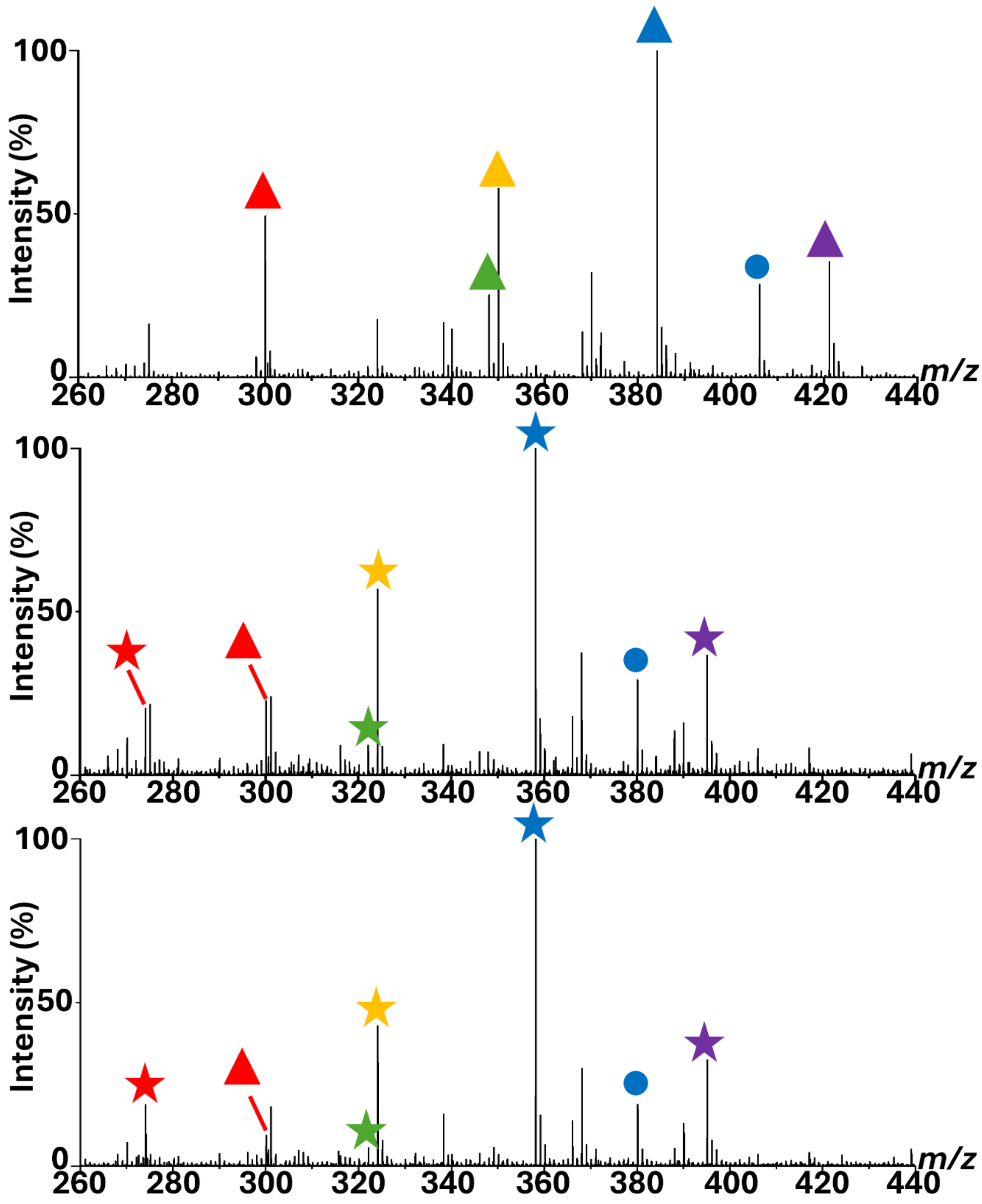

Figure S8 – LAP MALDI MS profiles of ampicillin (yellow), cefalexin (green), imipenem (red), doripenem (purple) and meropenem (blue) obtained from susceptible *K. pneumoniae* (top panel), susceptible *K. pneumoniae* spiked with penicillinase (middle panel), and resistant *K. pneumoniae* (bottom panel). The ▲ symbol indicates intact antibiotics. The ★ symbol indicates hydrolyzed decarboxylated antibiotic products. Additional sodiated antibiotic peaks are marked with •.

Supplementary Table

Table S1 – Theoretical and observed *m/z* values in LAP-MALDI MS profiles for the intact antibiotics and their hydrolyzed decarboxylated products, along with their respective ppm errors.

**Antibiotic**	**Observed *m/z* value**	**Theoretical *m/z* value**	**PPM**
Ampicillin $[M+H]^+$	350.1190	350.1175	4.28
Ampicillin $[M+H_2O-CO_2-H]^+$	324.1392	324.1382	3.09
Cefalexin $[M+H]^+$	348.1037	348.1018	5.46
Cefalexin $[M+H_2O-CO_2-H]^+$	322.1236	322.1225	3.41
Doripenem $[M+H]^+$	421.1234	421.1216	4.27
Doripenem $[M+H_2O-CO_2-H]^+$	395.1440	395.1423	4.30
Imipenem $[M+H]^+$	300.1032	300.1018	4.67
Imipenem $[M+H_2O-CO_2-H]^+$	274.1247	274.1225	8.03
Meropenem $[M+H]^+$	384.1609	384.1593	4.16
Meropenem $[M+H_2O-CO_2-H]^+$	358.1818	358.1801	4.75
